# Hierarchical Pt-In Nanowires for Efficient Methanol Oxidation Electrocatalysis

**DOI:** 10.3390/molecules28031502

**Published:** 2023-02-03

**Authors:** Zhao Lu, Lu Zou, Wulin Song

**Affiliations:** 1 Analytical and Testing Center of Huazhong University of Science and Technology, Wuhan 430074, China; 2 State Key Laboratory of Materials Processing and Die & Mould Technology, Huazhong University of Science and Technology, 1037 Luoyu Road, Wuhan 430074, China

**Keywords:** Pt-In nanowires, methanol oxidation, electrocatalysis

## Abstract

Direct methanol fuel cells (DMFC) have attracted increasing research interest recently; however, their output performance is severely hindered by the sluggish kinetics of the methanol oxidation reaction (MOR) at the anode. Herein, unique hierarchical Pt-In NWs with uneven surface and abundant high-index facets are developed as efficient MOR electrocatalysts in acidic electrolytes. The developed hierarchical Pt_89_In_11_ NWs exhibit high MOR mass activity and specific activity of 1.42 A mg_Pt_^−1^ and 6.2 mA cm^−2^, which are 5.2 and 14.4 times those of Pt/C, respectively, outperforming most of the reported MORs. In chronoamperometry tests, the hierarchical Pt_89_In_11_ NWs demonstrate a longer half-life time than Pt/C, suggesting the better CO tolerance of Pt_89_In_11_ NWs. After stability, the MOR activity can be recovered by cycling. XPS, CV measurement and CO stripping voltammetry measurements demonstrate that the outstanding catalytic activity may be attributed to the facile removal of CO due to the presence of In site-adsorbing hydroxyl species.

## 1. Introduction

Direct methanol fuel cells (DMFC) are a promising energy conversion device with several advantages, including high conversion efficiency, high portability and low pollutant emission, which have drawn much research interest [1,2,3,4]. Unfortunately, the methanol oxidation reaction (MOR) occurring at the anode of DMFC is very sluggish in kinetics, limiting the output performance of DMFC [3,5,6]. A large amount of electrocatalysts are required to accelerate the MOR rate. To date, commercial Pt/C, platinum nanoparticles (NPs) supported on carbon, is the most common anode catalyst for DMFC. Yet, Pt NPs will suffer from CO poisoning during the MOR process, where CO is a reaction intermediate and will block the active site of Pt NPs for further reaction, leading to a decay in activity [7,8]. Alternative electrocatalysts to Pt/C are highly sought after.

To address the effect of CO poisoning and improve the MOR activity of Pt-based electrocatalysts, several strategies have been proposed in the past few years. On the one hand, Pt can alloy with 3D transition metals (Ni, Co or Cu) to modulate the electronic structure of the Pt surface, which can weaken the binding strength between CO and Pt atoms and thus improve activity [9,10,11,12]. For example, Li et al. reported Cu-Ni@Pt-Cu core@shell NPs as efficient MOR catalysts with a high mass activity (MA) of 0.99 A mg_Pt_^−1^ [11]. Alternatively, Pt-Ni supported on graphene also displayed good MOR activity in previous reports [10,13]. Another effective approach is to introduce a second oxyphilic metal, such as Ru, In and Sn, to adsorb hydroxyl species and to oxidate the adsorbed CO, which is referred to as a bifunctional effect [14,15,16,17,18]. For example, Du et al. reported PdIn nanocrystals with coral-like morphology as efficient catalysts for MOR. The obtained PdIn nanocrystals demonstrated a high SA of 3.04 mA cm^−2^ [18]. Zerdoumi et al. found that the composition of In/Sn in Pd_2_In_1−x_Sn_x_ can modulate the reaction path, where MOR mainly proceeds via CO (indirect path) when 0 ≤ *x* < 0.8 and via formate (direct pathway) when 0.8 ≤ *x* ≤ 1 [19]. Meanwhile, engineering the surface structure and morphology, especially the structure of one-dimensional nanowires (NWs), can provide plenty of active sites for further activity enhancement [20,21,22]. In these regards, one promising strategy is to develop a Pt-based alloy with 1D architecture, so as to obtain good MOR activity.

In this work, we report the hierarchical Pt-In NWs with rough surface as efficient MOR electrocatalysts. We prepare hierarchical Pt-In nanowires via a facile wet-chemical approach. The obtained Pt-In NWs feature an uneven surface with abundant high-index facets. Among the obtained Pt-In NWs, Pt_89_In_11_ NWs demonstrate the best MOR activity, with high MOR MA and SA of 1.42 A mg_Pt_^−1^ and 6.2 mA cm^−2^, respectively. The enhanced MOR activity may be ascribed to the presence of the oxyphilic element In, which helps to remove CO at a lower potential.

## 2. Result and Discussion

Hierarchical Pt-In NWs were prepared by a wet-chemical co-reduction approach. Briefly, the Pt-In NWs were prepared in oleylamine (OAm) using Pt(acac)_2_ and In(acac)_3_ as metal precusors. CTAB was employed as a surfactant for the formation of NWs, while glucose was chosen as a reducing agent. After reacting at 180 °C in an oil bath for 6 h, Pt-In NWs were obtained.

**Figure 1:**
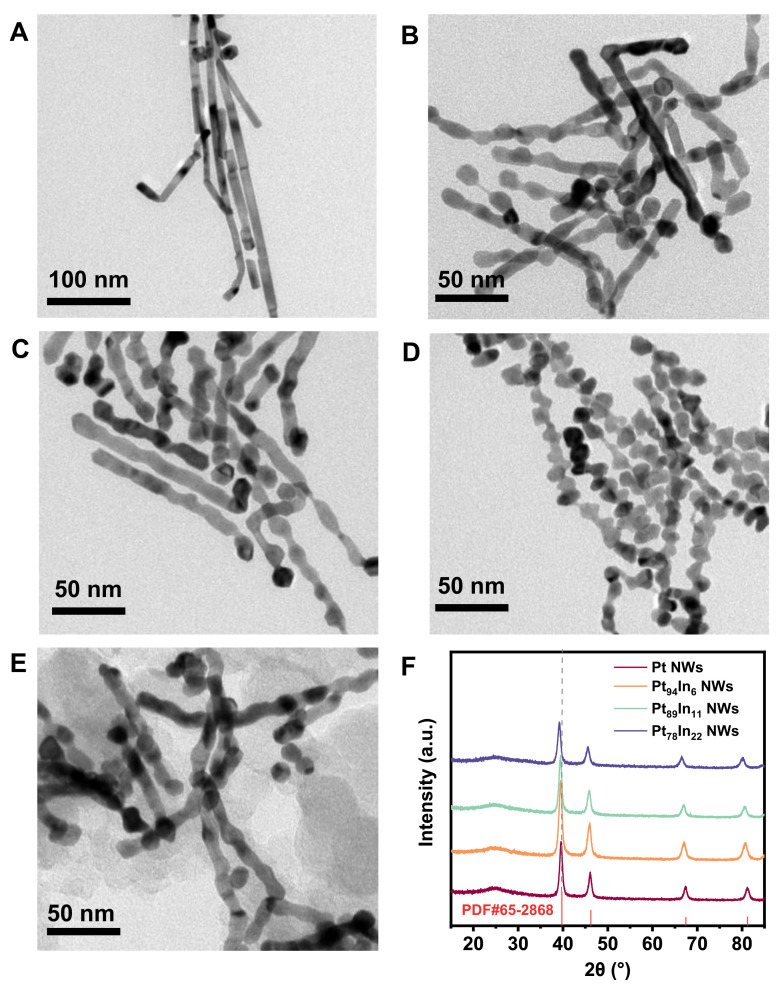
TEM images of (**A**) Pt NWs, (**B**) Pt_94_In_6_ NWs, (**C**) Pt_89_In_11_ NWs, (**D**) Pt_78_In_22_ NWs. (**E**) TEM image of carbon-supported Pt_89_In_11_ NWs. (**F**) XRD patterns of Pt and Pt-In NWs.

The morphology and structure of the product was characterized by transmission electron microscopy (TEM). Figure 1 shows the low-magnified TEM images of the obtained nanocrystals, where one-dimensional (1D) NWs are the main product. As shown in Figure 1A, the diameter of Pt NWs is about 10 nm, while the length reaches several hundred nanometers, bounded by a very smooth surface. After the introduction of the In element, the 1D morphology can be maintained. The diameter of Pt-In, with different compositions, is quite similar to that of Pt NWs (10 nm). Interestingly, the surface of the Pt-In NWs becomes rough and uneven with many periodic bumps on it, exhibiting a jewel-like hierarchical nanostructure. Such uneven and rough surfaces may provide a large amount of low-coordinated active sites, such as steps and corners, benefiting catalytic activity. The Pt/In ratios of three Pt-In NWs are determined by ICP-OES, which are about 94/6, 89/11 and 78/22, respectively, and very close to the feeding ratio. The NWs are loaded on commercial carbon for further use (Figure 1E and Figure S1). XRD was performed to characterize the crystal structure of the NWs. As shown in Figure 1D, all of the NWs show the typical face-centered cubic (FCC) structure, with (111), (200) and (220) facets located at around 40°, 46° and 67°, respectively. Furthermore, the diffraction peaks of Pt-In NWs show an obvious left-shift, which is ascribed to the introduction of In with a larger atomic radius.

**Figure 2:**
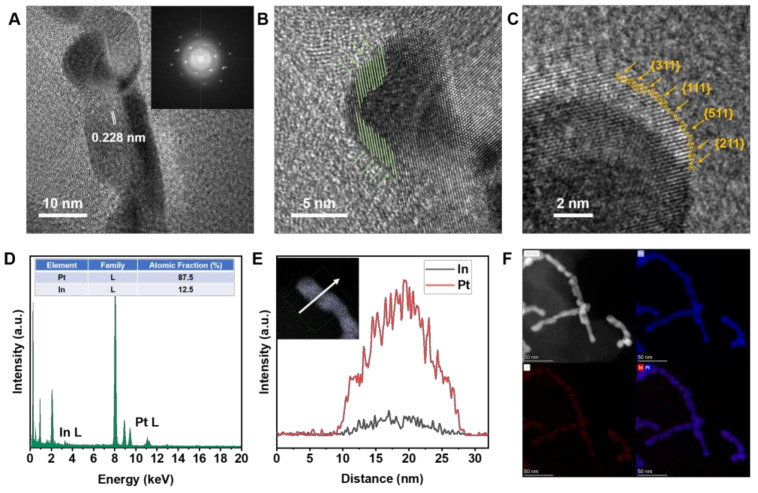
(**A**) HRTRM image, (**B**,**C**) atomic-resolution HRTEM images, (**D**) STEM-EDS, (**E**) SETM-EDS line scan profile, and (**F**) STEM-EDS elemental mappings of Pt_89_In_11_ NWs.

Figure 2A shows high-resolution TEM images of Pt_89_In_11_ NWs. The corresponding fast Fourier transform (FFT) pattern also confirms the quasi-single crystalline nature of Pt_89_In_11_ NWs. The spacing lattice fringe is measured to be 0.228 nm, corresponding to (111) facet of FCC Pt. Interestingly, when magnified to atomic resolution, an uneven surface can be clearly observed (Figure 2B,C). Some surface steps and concave sites can be identified by the arrow. In addition, plenty of high-index facets, such as {311}, {211} or even {511}, are clearly identified on the uneven surface of the hierarchical Pt_89_In_11_ NWs, which is beneficial to improving the intrinsic electrocatalytic activity [23,24]. The ratio of Pt/In is determined to be 87.5/12.5, as characterized by TEM-EDS analysis (Figure 2D), which is in accordance with the ICP-OES results. Moreover, the EDS line scan and elemental mapping results reveals that the Pt and In elements distribute uniformly in the whole NW.

**Figure 3:**
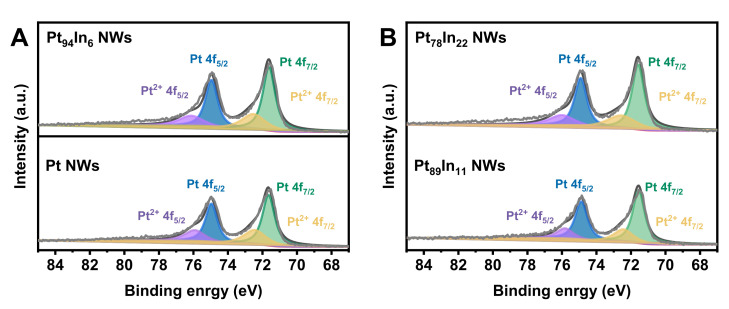
High-resolution Pt 4*f* XPS spectra of (**A**) Pt_96_In_6_ NWs and Pt, (**B**) Pt_78_In_22_ NWs and Pt_89_In_11_ NWs.

X-ray photoelectron spectroscopy (XPS) was carried out to analyze the valence state of Pt in Pt and Pt-In NWs (Figure S2). All the binding energies were calibrated with C 1 s at 284.8 eV as a reference. Figure 3A,B show the high-resolution Pt 4*f* XPS spectra. The binding energies at around 71.5 eV and 74.5 eV can be assigned to 4*f*_7/2_ and 4*f*_5/2_ orbitals of metallic Pt [25,26]. Meanwhile, the peaks at around 72.4 eV and 75.8 eV are associated with Pt (II). The Pt (0) to Pt (II) ratio is calculated to be ~2/1 for these four samples, indicating that Pt is mainly in a metallic state. In addition, the Pt 4*f*_7/2_ binding energies of Pt and Pt-In NWs are very close (~71.5 eV), which suggests a similar electronic structure.

**Figure 4:**
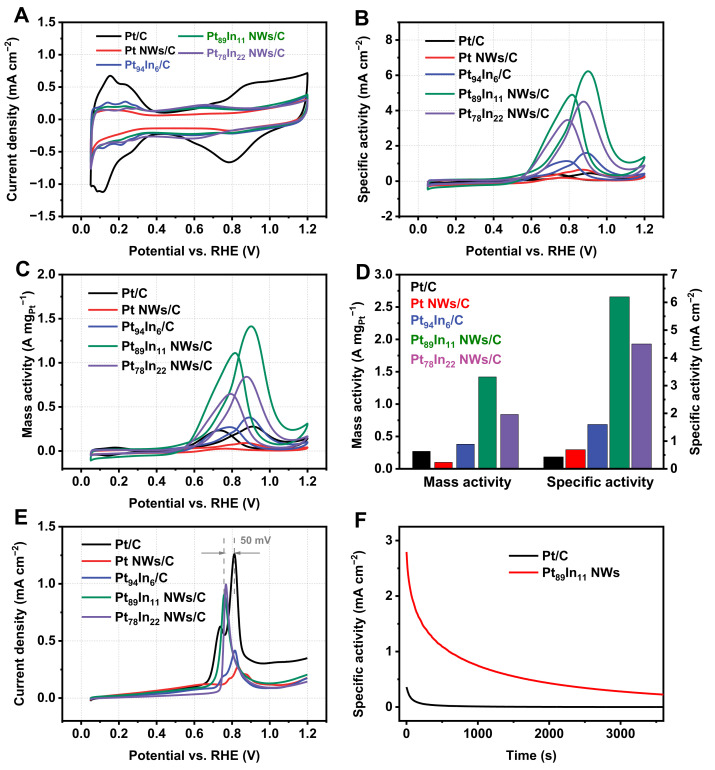
(**A**) CV curves of the studied electrocatalysts in 0.1 M HClO_4_. (**B**) ECSA normalized and (**C**) mass normalized CV curves of the studied electrocatalysts in 0.1 M HClO_4_ + 0.5M methanol. (**D**) Comparison of MA and SA of the studied electrocatalysts. (**E**) CO stripping curves of the studied electrocatalysts. (**F**) I-t curves of Pt/C and Pt_89_In_11_ NWs.

The obtained Pt-In NWs were then employed as MOR electrocatalysts, and commercial Pt/C (60 wt.%) was used as a reference. Figure 4A shows the CV curves of the studied electrocatalysts in N_2_-saturated 0.1 M HClO_4_. Three distinct regions can be observed, including underpotential adsorption and desorption of hydrogen (H_upd_) at the potential range of 0–0.4 V, double-layer region, and surface adsorption/reduction of hydroxyl species on Pt surface (above 0.7 V). An interesting phenomenon is that after introducing the In element, the surface adsorption of the hydroxyl species appears at a relatively low potential (~0.65 V), which can be attributed to the strong oxyphilicity of the In element. Therefore, we hypothesize that the presence of hydroxyl species will benefit the oxidation of CO species and improve the MOR activity. Further evidence, such as in situ Raman spectroscopy, will be carried out to verify this hypothesis. The electrochemical surface areas (ECSA) of the studied electrocatalysts are 62.3, 14.0, 23.8, 22.7 and 18.7 m^2^ g_Pt_^−1^ for Pt/C, Pt NWs, Pt_94_In_6_ NWs, Pt_89_In_11_ NWs, and Pt_78_In_22_ NWs, respectively, calculated according to H_upd_.

We measured the MOR activity of the obtained electrocatalysts in N_2_-saturated 0.1 M HClO_4_ + 0.5 M methanol. Figure 4B,D show the MOR specific activity (SA) and MA of the studied electrocatalysts. Specifically, the MA and SA of Pt_89_In_11_ NWs reach 1.42 A mg_Pt_^−1^ and 6.2 mA cm^−2^, which are 5.2 and 14.4 times those of Pt/C, respectively (Figure 4D). The superior MOR activity of the Pt_89_In_11_ NWs represents one of the most efficient Pt alloy MOR catalysts evaluated in recent reports (Table S1). We also measured the MOR activity of Pt-In NWs with different Pt/In ratios. A volcano relationship is observed between the MOR activity and Pt/In ratio, where Pt_89_In_11_ NWs demonstrate the best MOR activity when compared to Pt_94_In_6_ NWs and Pt_78_In_22_. An appropriate Pt/In ratio is essential to balance the adsorption and desorption of reaction intermediates to accelerate the reaction rate according to the Sabatier principle [5].

CO stripping tests were performed to further evaluate the CO tolerance of Pt-In NWs. As shown in Figure 4E, the CO stripping peak potential of Pt_89_In_11_ NWs is 50 mV more positive than that of Pt/C, which suggests the facile CO oxidation of Pt_89_In_11_ NWs. Combining with CV observations, the introduction of In can promote the adsorption of hydroxyl species, which can assist the oxidation of a CO intermediate. The facile removal of CO can expose fresh Pt active sites for MOR, thus enhancing the MOR activity.

Electrochemical durability was evaluated by a chronoamperometry (CA) test of 3600 s. As shown in Figure 4F, Pt_89_In_11_ NWs still have an SA of 0.27 mA cm^−2^, while Pt/C nearly decays to 0 mA cm^−2^. The half-life time of Pt_89_In_11_ NWs in the CA test is 280 s, much longer than that of Pt/C (54 s), which suggests that the Pt_89_In_11_ NWs demonstrate better CO tolerance. Meanwhile, CV tests were performed after CA stability tests (Figure S3). After re-activation by CV scan, the MOR activity of Pt_89_In_11_ NWs can nearly recover to the initial activity, suggesting the high stability. In addition, we also characterized the change in morphology and composition of Pt_89_In_11_ NWs (Figure S4). Notably, the Pt_89_In_11_ NWs show negligible change after the stability test, further suggesting the good stability.

## 3. Experimental Section

### 3.1. Chemicals

Platinum (II) acetylacetonate (Pt(acac)_2_, 97%), In(acac)_3_ (99%), glucose (≥99.5%), Cetyltrimethylammonium boride (CTAB, AR) and oleylamine (OAm, 80–90%) were purchased from Aladdin reagent. Commercial Pt/C (60%) were obtained from Johnson Matthey. All reagents were used without any further purification. The deionized water (18.2 MΩ·cm^−1^) was used all through the experiments.

### 3.2. Characterization

Transmission electron microscopy (TEM) was performed on Tecnai G20 (FEI, Netherlands) at an operation voltage of 200 kV. High-resolution TEM (HR-TEM) and high-angle annular dark-field scanning TEM (HAADF-STEM) images were obtained from Talos F200X (FEI, Czech Republic) working at 200 kV. X-ray diffraction (XRD) patterns were recorded on a Bragg-Brentano diffractometer (D8-tools, Germany) equipped with a Cu Kα-emitting source (*λ* = 1.5418 Å). X-ray photoelectron spectroscopy (XPS) analysis was carried out on an Axis Supra+ instrument (Shimadzu-Kratos, Japan). Inductively coupled plasma mass spectrometry (ICP-OES) tests were measured on an ELAN 9000/DRC ICP-OES system.

### 3.3. Preparation of Hierarchical Pt-In NWs

In a typical synthesis of hierarchical Pt_89_In_11_ NWs, 10 mg Pt(acac)_2_, 1 mg In(acac)_3_, 50 mg CTAB and 60 mg glucose were mixed with 5 mL oleylamine in a vial. The mixture was vigorously sonicated for 30 min to obtain a homogeneous solution. The vial was then placed in an oil bath and the temperature increased from 30 to 180 °C within 30 min. The reaction was maintained at this temperature for another 6 h before it was gradually cooled to room temperature. The as-prepared NWs were collected by centrifugation (8000 rpm, 3 min) and washed with a hexane/ethanol mixture three times. The preparation of Pt and other Pt-In NWs was similar, except for changing the feeding amount of In(acac)_3_. The obtained NWs were again dispersed in hexane for further use.

### 3.4. Loading the Obtained NWs onto Carbon

An amount of 12 mg of Vulcan XC-72 carbon powder was first dispersed in an isopropanol/hexane/acetate acid mixture (*v/v/v* 10/10/1) by sonication. Afterwards, the obtained NWs in hexane were added dropwise to the above carbon suspension. After sonication for another 60 min, the product was collected by centrifugation at 9000 rpm and washed three times with ethanol. The final product was dried in an oven at 60 °C under vacuum.

### 3.5. Electrochemical Measurements

Electrochemical tests were performed on a CHI760E electrochemical station (Shanghai Chenhua Co., Shanghai, China). Glassy carbon (5 mm in diameter), Ag/AgCl electrode (saturated KCl), were employed as the working electrode, reference electrode and counter electrode, respectively. All potentials were converted to values with reference to a reversible hydrogen electrode (vs. RHE).

The electrocatalyst ink was prepared as follows: 1 mg electrocatalysts was mixed with isopropanol and Nafion (5%) (*v/v* = 1:0.1, 1 mg_catal_ mL^−1^) by sonication for 30 min. Then, 10 μL of ink was cast on the working electrode and dried under ambient conditions. The final loading of Pt was determined by ICP-OES.

All of the electrochemical tests were carried out at room temperature. Cyclic voltammetry (CV) curves were obtained in N_2_-saturated 0.1 M HClO_4_ solutions at a scan rate of 50 mV s^−1^ between 0–1.2 V (vs. RHE). MOR measurements were conducted in N_2_-saturated 0.1 M HClO_4_ + 0.5 M methanol at a scan rate of 50 mV s^−1^. The stability of the catalyst was evaluated by chronoamperometry at 0.8 V vs. RHE for 3600 s.

For CO-stripping tests, CO gas (99.9%) was first bubbled into 0.1 M HClO_4_ for 10 min while the working electrode was holding at 0.1 V at the same time. Then, the electrolyte was purged with N_2_ for 15 min while the working electrode was holding at 0.1 V. Afterwards, two CVs (0–1.0 V vs. RHE) were recorded at a scan rate of 20 mV s^−1^.

## 4. Conclusions

In conclusion, we have successfully synthesized unique hierarchical Pt-In NWs with uneven surfaces as efficient MOR electrocatalysts in an acidic electrolyte. The developed hierarchical Pt_89_In_11_ NWs exhibit high MOR MA and SA of 1.42 A mg_Pt_^−1^ and 6.2 mA cm^−2^, which are 5.2 and 14.4 times those of Pt/C, respectively. The hierarchical Pt_89_In_11_ NWs also display a respectable stability, with negligible performance decay after CA test. The impressive MOR activity make our hierarchical Pt_89_In_11_ NWs one of the best MOR catalysts among the reports. According to XPS, CV measurement and CO stripping voltammetry, the outstanding catalytic activity can be attributed to the presence of an In site adsorbing hydroxyl species to oxidate CO species instead of the modified electronic structure. This work demonstrates a promising strategy for shape- and structure-controlled electrocatalysts with advanced activity and durability, which will be of great significane for energy conversion applications and beyond.

## Data Availability

Not applicable.

## Supplementary Materials

The following supporting information can be downloaded at: https://www.mdpi.com/article/10.3390/molecules28031502/s1, Figure S1: TEM images of carbon-supported (A) Pt NWs, (B) Pt_94_In_6_ NWs, (C) Pt_89_In_11_ NWs and (D) Pt_78_In_22_ NWs; Figure S2: XPS survey spectra of Pt and Pt-In NWs; Figure S3: I-t curves of Pt/C and Pt_89_In_11_ NWs; Figure S4: (A) TEM image and (B) TEM-EDS profile of Pt_89_In_11_ NWs after stability test; Table S1: MOR activity of Pt alloy catalysts in recent reports. References [27,28,29,30,31,32,33,34,35,36,37,38] are cited in the Supplementary Materials.
