# Supplementary material for: Hierarchical Pt-In Nanowires for Efficient Methanol Oxidation Electrocatalysis
Source: Molecules. 2023 Feb 3;28(3):1502. doi: 10.3390/molecules28031502 (PMC9920629; doi:10.3390/molecules28031502)
Supplement: Supplementary file 1 [file molecules-28-01502-s001.zip › molecules-2112305-supplementary.pdf]

Hierarchical Pt-In Nanowires for Efficient Methanol Oxidation Electrocatalysis

Zhao Lu [1], Lu Zou [2] and Wulin Song [1,2,*]

[1] Analytical and Testing Center of Huazhong University of Science and Technology, Wuhan 430074, China
[2] State Key Laboratory of Materials Processing and Die & Mould Technology, Huazhong University of Science and Technology, 1037 Luoyu Road, Wuhan 430074, China
* Correspondence: wulins@126.com; Tel.: +86-027-875592025

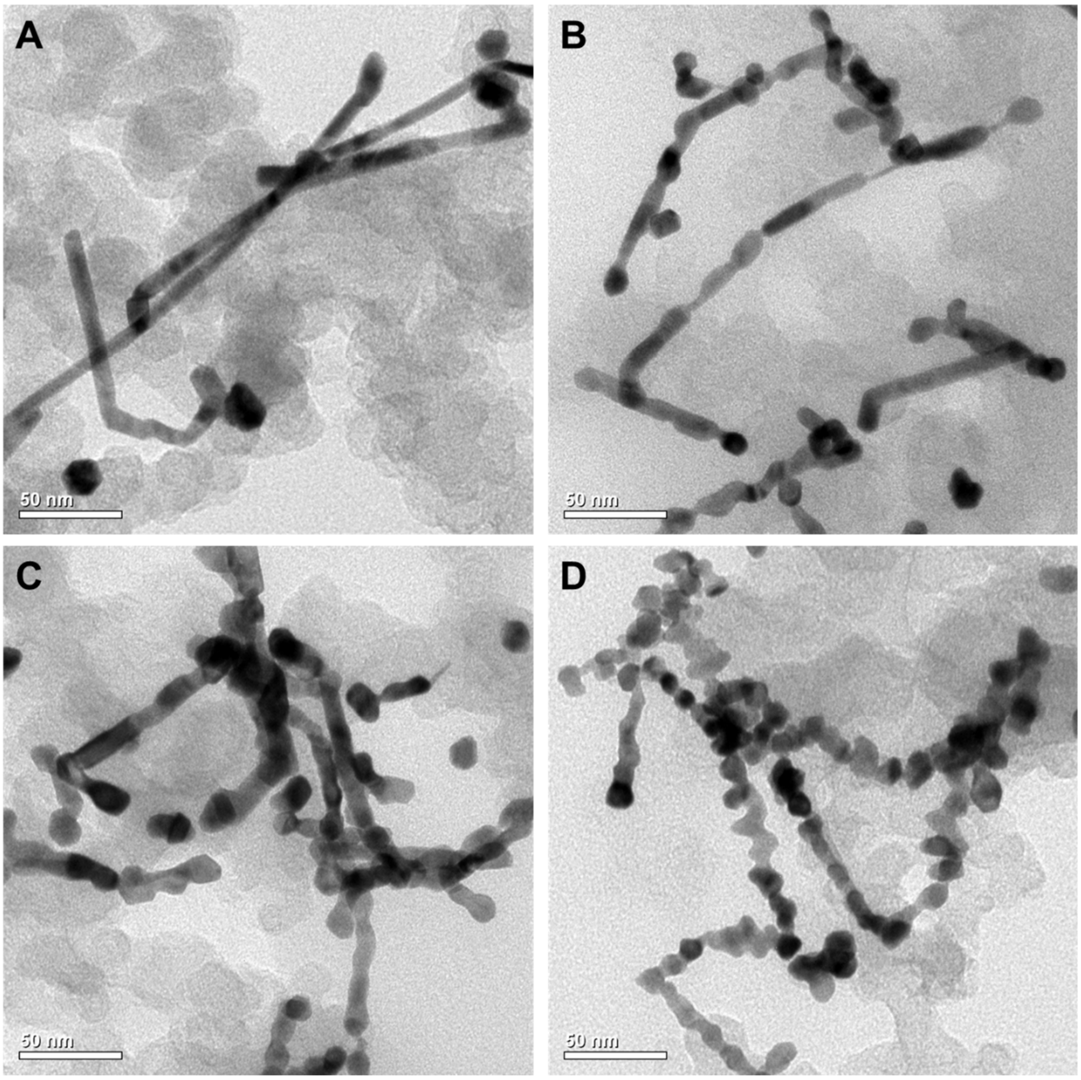

Figure S1. TEM images of carbon supported (**A**) Pt NWs, (**B**) $Pt_{94}In_6$ NWs, (**C**) $Pt_{89}In_{11}$ NWs, and (**D**) $Pt_{78}In_{22}$ NWs.

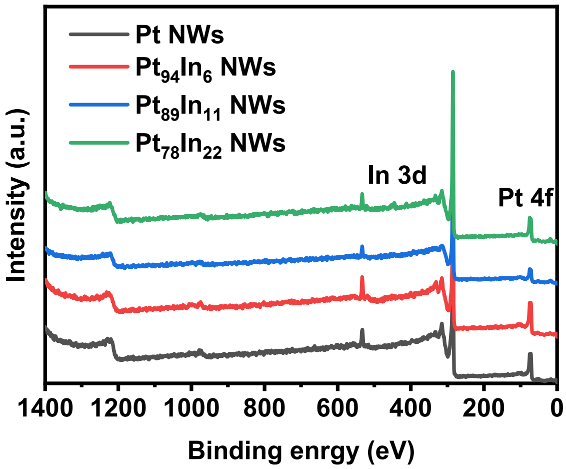

Figure S2. XPS survey spectra of Pt and Pt-In NWs.

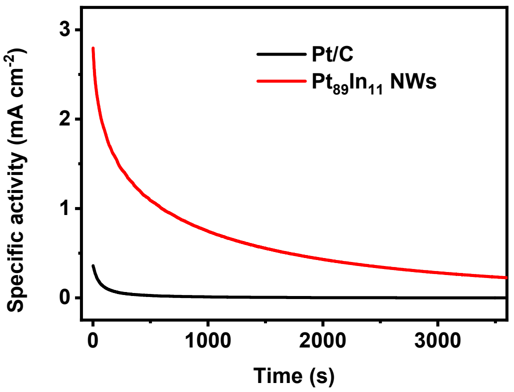

Figure S3. I-t curves of Pt/C and $Pt_{89}In_{11}$ NWs.

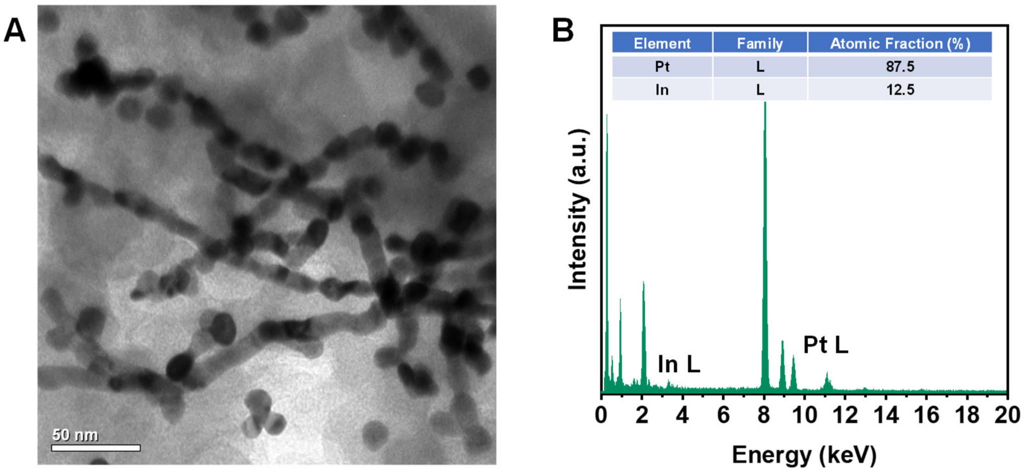

Figure S4. (**A**) TEM image, and (**B**) TEM-EDS profile of $Pt_{89}In_{11}$ NWs after stability test.

Table S1. MOR activity of Pt alloy catalysts in recent reports.

Catalyst	MA (A mg^{-1}_{Pt})	SA (mA cm^{-2})	Electrolytes	Ref.
**$Pt_{89}In_{11}$ NWs**	**1.42**	**6.2**	**0.1 M $HClO_4$ + 0.5 M Methanol**	**This Work**
Pt_7Ru_2Fe Nanowires	NA	2.27	0.1 M $HClO_4$ + 0.5 M Methanol	[1]
Pt Nanosheets	0.27	0.57	0.5 M H_2SO_4 + 1 M Methanol	[2]
PtPb/Pt Nanoplates/C	1.50	2.70	0.1 M $HClO_4$ + 0.1 M Methanol	[3]
$PtCu_{2.1}$ Nanowires/C	1.56	3.31	0.1 M $HClO_4$ + 0.2 M Methanol	[4]
PtRu Nanowires/C	0.82	1.16	0.1 M $HClO_4$ + 0.5 M Methanol	[5]
PtRu Nanodendrites	1.08	10.08	0.1 M $HClO_4$ + 1 M Methanol	[6]
Co-PtCu Nanoframes/C	4.11	7.09	0.1 M $HClO_4$ + 1 M Methanol	[7]
PtRuCu Hexapods	1.35	3.91	0.1 M $HClO_4$ + 1 M Methanol	[8]
$Pd_{0.97}Ru_{0.44}Pt$ Nanowire Networks	1.10	1.98	0.1 M $HClO_4$ + 0.5 M Methanol	[9]
PtCoNiRh Nanowires/C	1.36	2.08	0.1 M $HClO_4$ + 0.5 M Methanol	[10]
f-Pt_2Cu_1 Nanowires	1.14	2.78	0.1 M $HClO_4$ + 0.5 M Methanol	[11]
Pt_3Sn Intermetallic Nanocubes	2.19	4.00	0.1 M $HClO_4$ + 1 M Methanol	[12]

References:

1. Scofield, M.E.; Koenigsmann, C.; Wang, L.; Liu, H.; Wong, S.S. Tailoring the composition of ultrathin, ternary alloy PtRuFe nanowires for the methanol oxidation reaction and formic acid oxidation reaction. *Energ Environ. Sci*. **2015**, *8*, 350–363.
2. Chhetri, M.; Rana, M.; Loukya, B.; Patil, P.K.; Datta, R.; Gautam, U.K. Mechanochemical Synthesis of Free-Standing Platinum Nanosheets and Their Electrocatalytic Properties. *Adv. Mater*. **2015**, *27*, 4430–4437.
3. Bu, L.; Zhang, N.; Guo, S.; Zhang, X.; Li, J.; Yao, J.; Wu, T.; Lu, G.; Ma, J.-Y.; Su, D.; et.al Biaxially strained PtPb/Pt core/shell nanoplate boosts oxygen reduction catalysis. *Electrocatalysis*. **2016**, *354*, 1410–1414.
4. Zhang, N.; Bu, L.; Guo, S.; Guo, J.; Huang, X. Screw Thread-Like Platinum-Copper Nanowires Bounded with High-Index Facets for Efficient Electrocatalysis. *Nano Lett*. **2016**, *16*, 5037–5043.
5. Huang, L.; Zhang, X.; Wang, Q.; Han, Y.; Fang, Y.; Dong, S. Shape-Control of Pt-Ru Nanocrystals: Tuning Surface Structure for Enhanced Electrocatalytic Methanol Oxidation. *J. Am. Chem Soc*. **2018**, *140*, 1142–1147.
6. Lu, S.; Eid, K.; Ge, D.; Guo, J.; Wang, L.; Wang, H.; Gu, H. One-pot synthesis of PtRu nanodendrites as efficient catalysts for methanol oxidation reaction. *Nanoscale*. **2017**, *9*, 1033–1039.
7. Kwon, T.; Jun, M.; Kim, H.Y.; Oh, A.; Park, J.; Baik, H.; Joo, S.H.; Lee, K. Vertex-Reinforced PtCuCo Ternary Nanoframes as Efficient and Stable Electrocatalysts for the Oxygen Reduction Reaction and the Methanol Oxidation Reaction. *Adv. Funct Mater*. **2018**, *28*, 1706440.
8. Xue, S.; Deng, W.; Yang, F.; Yang, J.; Amiinu, I.S.; He, D.; Tang, H.; Mu, S. Hexapod PtRuCu Nanocrystalline Alloy for Highly Efficient and Stable Methanol Oxidation. *ACS Catal*. **2018**, *8*, 7578–7584.
9. Shang, C.; Guo, Y.; Wang, E. Facile fabrication of PdRuPt nanowire networks with tunable compositions as efficient methanol electrooxidation catalysts. *Nano Res*. **2018**, *11*, 4348–4355.
10. Wang, W.; Chen, X.; Zhang, X.; Ye, J.; Xue, F.; Zhen, C.; Liao, X.; Li, H.; Li, P.; Liu, M.; et.al Quatermetallic Pt-based ultrathin nanowires intensified by Rh enable highly active and robust electrocatalysts for methanol oxidation. *Nano Energy*. **2020**, *71*, 104623.
11. Wang, K.; Huang, D.; Guan, Y.; Liu, F.; He, J.; Ding, Y. Fine-tuning the electronic structure of dealloyed PtCu nanowires for efficient methanol oxidation reaction. *ACS Catal*. **2021**, *11*, 14428–14438.

12. Chen, H.-S.; Benedetti, T.M.; Lian, J.; Cheong, S.; O'Mara, P.B.; Sulaiman, K.O.; Kelly, C.H.W.; Scott, R.W.J.; Gooding, J.J.; Tilley, R.D. Role of the Secondary Metal in Ordered and Disordered Pt-M Intermetallic Nanoparticles: An Example of Pt_3Sn Nanocubes for the Electrocatalytic Methanol Oxidation. *ACS Catal*. **2021**, *11*, 2235–2243.
